# Evaluation of the 3 mm Thickness Splint Therapy on Temporomandibular Joint Disorders (TMDs)

**DOI:** 10.1155/2018/3756587

**Published:** 2018-12-05

**Authors:** Nihat Akbulut, Ahmet Altan, Sibel Akbulut, Cemal Atakan

**Affiliations:** ^1^Associate Professor, Department of Oral and Maxillofacial Surgery, Faculty of Dentistry, Gaziosmanpaşa University, Tokat, Turkey; ^2^Assistant Professor, Department of Oral and Maxillofacial Surgery, Faculty of Dentistry, Gaziosmanpaşa University, Tokat, Turkey; ^3^Assistant Professor, Department of Orthodontics, Faculty of Dentistry, Gaziosmanpaşa University, Tokat, Turkey; ^4^Professor, Department of Statistics, Faculty of Sciences, Ankara University, Ankara, Turkey

## Abstract

*Objective*. This study aimed at finding out whether the 3 mm thickness of stabilization splints has positive or negative effects on all temporomandibular disorder (TMD) symptoms. *Materials and Methods*. The statistical calculation included 25 (22 females; 3 males) TMD patients who received 3 mm thickness stabilization splint therapy. They were evaluated according to follow-up treatment period, TMD pain, muscle pain, mouth opening, diet score, and splint usage time per day. *Results*. There was important treatment success that 22 (88%) of patients were totally healed. There was not any remarkable effect or advancement of splints on total healings of TMDs in first 3 months' period (11/25 patients, 44%). The mouth opening mean reached 38, 67 mm at 6 months and 41 mm at 12 months with remarkable success. Except one (4%) patient, other 24 (96%) patients had a normal diet score of 3 at the end of splint therapy. There was no correlation between splint usage duration a day and total healing of TMDs. *Conclusion*. We conclude that 3 mm splint therapy should maintain at least 6 months to achieve remarkable results. Splint should be used at least 12 h a day consistent with our results. Finally, diet score should be incorporated with TMD pain and amount of mouth opening; hence, we advise to use in one term as “total healing.”

## 1. Introduction

Temporomandibular disorders (TMDs) encompass internal derangements of the temporomandibular joint (TMJ), abnormalities of masticatory muscles and the neighboring structure of the TMJ, and TMJ-related headache conditions [1, 2]. In all manifestations of TMDs, the major negative effects the patients experience include jaw movement limitations and of course slight to severe pain in the head and neck regions [3]. TMDs include TMJ and facial pain, including tenderness to touch the facial region muscle (particularly masticatory muscles and the TMJ), uncoordinated jaw movements, and the presence of joint noise [4]. While many research studies have evaluated diet intake problems during postop patient follow-ups of TMD-related surgeries, some studies have also considered diet intake before and after treatment of both nonsurgical and surgical evaluations of TMD patients in the context of jaw movement and the level of pain the patient experienced [5–7].

Despite the use of various types of occlusal splints made from a range of soft and hard materials, the most common splint used to treat TMDs, the stabilization splint, remains a valid option highly agreed upon among practitioners [8]. According to the literature review, splints of various thicknesses from 1 to 15 mm have been used to treat TMDs, but a thickness of 3–5 mm is preferred along with comfortable alternatives [8].

Stabilization splints usually decrease TMD symptoms of pain from internal derangements or of myofascial origin to improve jaw movement and general health. In addition, they increase diet scores and improve disc displacement without reduction through splint therapy (e.g., individuals who could only eat liquid diets before can begin eating normal diets including solid food after splint therapy) [5–7].

This study aims to determine whether the 3 mm thickness of stabilization splints has positive or negative effects on disc displacement with or without reduction and TMDs symptoms (pain, muscle tenderness or pain, jaw movement, low diet score, and total healing) according to a range of follow-up periods (3, 6, and 12 months), splint usage time per day (hours), and demographic features of patients.

## 2. Materials and Methods

This study was conducted using the files of patients who received 3 mm stabilization splint therapy as a conservative or initial treatment of TMDs at the Gaziosmanpasa University Oral and Maxillofacial Surgery Clinic in Tokat, Turkey. The patients' files were from 2013 to 2017. TMDs were diagnosed in the same clinic according to the clinical and radiological data. Magnetic resonance imaging, the most accurate radiological tool, was used for diagnosis before initial treatments, and examinations were done based on *Diagnostic Criteria for Temporomandibular Disorders* (DC/TMD) axis I (updated by Schiffman et al. [2] in 2014), which is the main guide for evaluating patients to determine final diagnosis and treatment progress for TMDs. In the same clinic, informed consent was obtained from patients before beginning treatments, in which they consented to the use of their diagnosis and treatments as scientific data. This study was approved by the local ethics committee.

### 2.1. Data Variables of Patients

Twenty-five TMD patients (22 females, 3 males; 19–42 years old with a mean age of 30.52 years) received stabilization splint therapy with 3 mm thick splints and were evaluated during the treatment follow-up at 0, 3, 6, and 12 months for the following variables: disc displacement with or without reduction, TMD pain, muscle pain, jaw movement, diet score, splint usage time per day, treatment outcomes, and demographic data. The exclusion criteria for the study included the following:

- Calibration fault during the manufacturing of the splints detected from the patient files
- No regular follow-up visits or use of different splints or arthrocentesis for TMD treatment before starting the splint therapy with 3 mm thick splints
- The presence of systemic illness or anatomic conditions contributing to patients' TMDs (the presence of polyarthritis or other rheumatic disorders), and radiological findings of organic disease including TMJ detected

#### 2.1.1. Evaluation of Variables

1. TMD pain: pain was evaluated using the Verbal Analogue Scale (VAS) with 0 indicating no pain, 1 for mild pain, 2 for severe pain, and 3 for the highest level of pain.
2. Muscle pain: masticatory muscles including temporalis, masseter, pterygoid medial, and lateral were evaluated. If pain was present during a 5 second, 1 kilogram palpation of at least one muscle (according to DC/TMD) [2], 1 indicated yes (presence of pain) and 0 indicated no (no pain).
3. Disc displacement with or without reduction evaluation: before treatment, MRI and clinical examinations were done; during the follow-up period, only the clinical examinations were done. In addition, disc displacement parameters were evaluated with opening amount by the end of the treatment period.
4. Mouth opening amount: the degree of jaw movement was measured using an interincisal caliper (mm).
5. Diet score: this was evaluated using the VAS with 0 indicating a liquid-only diet, 1 for a soft diet, 2 for soft solids diet, and 3 for a standard diet with no limitations (the scoring system adapted or modified from Leandro et al. [7]).
6. Splint usage time per day (measured by hour, h): this was determined by the number of hours the splints had been used during the night or plus night during the day (24 h).
7. Treatment outcome: improvements of TMD pain, mouth opening, and diet score data were considered to determine whether or not an outcome of “total healing” was achieved. A score of 1 indicated total healing; 0 indicated a lack of total healing. In the event that a “0” score was determined, the patient was recommended for advanced treatment of TMDs.

### 2.2. Statistical Analysis

IBM SPSS Statistics for Windows, version 20.0 (IBM Corp, Armonk NY, 10504, USA) was used for statistical analysis of the collected data. To compare the two independent groups, independent two-sample *t*-test was used. When more than two independent groups were analyzed, the analysis of variance (ANOVA) test was used. In addition, for cross tabulation, the Fisher's exact test and chi-squared test both were used for checking with each other outcome. We considered the level of significance to be 5% (*p* ≤ 0.05).

## 3. Results

### 3.1. Outcomes of Variable Evaluations

Follow-up treatment period: 12 patients were followed up after 3 and 6 months and treated at the same time; 13 patients followed up after 3, 6, and 12 months exhibited full healing except 3 patients who had not completely healed during this period were advised to pursue advanced treatment modalities (Tables 1 and 2).

1. TMDs pain: patients' pain scores for the TMJ region were acquired before treatment and also the data in period of treatment were collected as well. By the end of 12 months, 84% of patients were pain-free. A remaining 16% of patients experienced only light pain after 12 months.  Table 3 shows the additional data regarding TMD pain.
2. Muscle pain: before splint therapy, 13 patients (52%) experienced muscle pain. By the end of 12 months of splint therapy, all except 4 patients (31%) had been treated successfully to eliminate muscle pain. Additional results are presented in Table 3.
3. Disc displacement with or without reduction evaluation: before treatment, 12 patients had disc displacement without reduction, and 13 patients had disc displacement with reduction. By the end of treatment period, remarkable outcome was achieved and is presented in Table 4.
4. Mouth opening amount: at the beginning of therapy, 12 patients (48%) had limited mouth opening ability (patients with disc displacement without reduction had a mean value of 32; 33 mm mouth opening). By the end of treatment period, the mouth opening mean reached 41 mm. This shows that 3 mm splint therapy may be the best choice for correcting mouth opening limitations in patients with disc displacement without reduction (Table 4).
5. Diet score: at the beginning of splint therapy, 3 patients (12%) were able to eat normal diets. After twelve months of splint therapy, all, except 1 patient (4%) who remained on a soft solid diet, were able to eat a normal diet, accounting for 24 patients (96%) (Table 1).
6. Splint usage time per day: there is no correlation or statistical significance concerning the duration of daily splint usage in relation to total healing (Table 2).
7. Treatment outcomes: twelve patients completed splint therapy with total healing at 6 months. The remaining 13 patients continued splint therapy through 12 months, and 10 of which attained an outcome of total healing. The remaining 3 patients (12%) had been advised to pursue advanced surgical treatment (Table 5).

## 4. Discussion

Despite many treatment difficulties for patients with TMDs, to date 90% of patients who have been treated for symptoms (e.g., pain, restriction of mouth opening and food intake, or low diet scores [5]) no longer experienced these symptoms after using conservative options such as splint therapy. Only 10% of TMD patients tend to have the need of advanced treatment alternatives, such as arthroscopic or arthrocentesis lysis and lavage and open surgery modalities [9]. The usage of occlusal splints as an early or conservative treatment modality for TMD patients is currently a very common clinical practice [3, 4, 10–13]. Of the many types of manufactured splints used, occlusal stabilization splints made of rigid acrylic and manufactured in contact with all mandibular teeth occlusal surface are preferred for these kinds of conservative treatments according to the literature [4, 8, 11, 13, 14]. Aside from a few sources [8, 15, 16], research has not extensively covered the effects of splint thickness. In this study, we focused our research on the effectiveness of splints with a 3 mm thickness to treat TMD because of positive reports from Lin et al. [8] and Abekura et al. [15] that found 3 mm splints to be superior in comfort and usage to 5 mm and 6 mm splints, which had the worst effects. Moreover, Hegab et al. [16] concluded in their study that 4 mm splints were nearly as effective as 3 mm splints on disc displacement with reduction, and in contrast to our study, 6 mm splints were only found to be effective on disc displacement without reduction. Pita et al. [17] reported that 3 and 6 mm splints were both effective in treating TMJ-related muscle disorders while our study demonstrated that 3 mm splints may be preferable over 6 mm splints due to comfort of use.

The most important result of our study is that the duration of daily splint usage has no correlation with or statistical significance to the total healing (defined by the improvement or healing of all TMDs symptoms, including pain, mouth opening limitation, and diet score; *p*=0.450 > 0.05 at 3 months, *p*=0.154 > 0.05 at 6 months, and *p*=0.309 > 0.05 at 12 months) process of 3 mm splint treatment of TMD patients. We only suggested that patients use splints for at least 12 hours per day without providing any other counseling, leading patients to use splints for 8 to 20 hours per day. Prior research suggests that stabilization splints should be used at night to meet the necessary daily usage time [3, 4, 8, 18]. De Rossi et al. [19] warn against continuous usage of splints, which could cause irreversible damage to occlusal relations and also suggest usage of the splint at night unless there is tooth clenching when driving or exercising, which could be rectified by increasing splint usage during these times. The results of our study align with reports from Davies and Gray [20] that there is no statistically significant advantage to any pattern of splint usage for 24 hours per day or only during the day or night. Similar to our study that we suggested to our patient to use their splint at least 12 h at night or plus day time, Badel et al. [3] and Conti et al. [4] instructed their patients to use splints at night, and their patients had successful treatment outcomes with high satisfaction. In contrast to our study, Kurita et al. [21] instructed their patients to use splints 24 hours per day, and they concluded that splint therapy had a 60–70% success rate treating symptoms such as TMJ pain, maximal mouth opening, and masticatory muscle pain, with a poor success rate for TMJ noise and disk derangement symptoms. They provided no conclusions about permanent TMJ damage due to the 24-hour continuous use of the splint.

Alencar and Becker [10] instructed their patients to use their splints for 24 hours per day in the first week and to use them only during the day for the following weeks to prevent permanent TMJ damage. Moreover, Kuzmanovic Pficer et al. [22] reported that their patients had been instructed to use their splints continuously (24 hours per day) and claimed that the jaw position resulted in occlusal stability. In contrast to Kuzmanovic Pficer et al. [22], Klasser and Greene [13] reported that TMD treatment is conservative and reversible as long as patients avoid full-time wear that can lead to permanent occlusal changes; the worst-case treatment outcome should be no worse than a failure to relieve symptoms. Similar to Klasser and Greene's approach, in our study, we instructed patients to use their splints for a minimum of only 12 hours per day to avoid permanent damage to TMJ structures.

Much of the available literature [3, 4, 8, 10, 18] overlooks the issue of when practitioners or patients should end splint therapy. For how many months should practitioners extend treatment: 3, 6, 12, or more? Existing literature does not appear to reach a consensus on this issue. Alencar and Becker [10] and Proff et al. [18] prescribed splint therapy to their patients for 3 months; Badel et al. [3] and Conti et al. [4] extended therapy to 6 months; finally Lin et al. [8] prescribed 12 months of therapy. In addition, Kuzmanovic Pficer et al. [22] reported that short-term (for about 3 months) and long-term (6 to 12 months) splint therapy had the same effect on TMD symptoms. In contrast to Kuzmanovic Pficer et al. [22], our splint therapy did not yield remarkable success for TMD symptoms in the short term. We followed up in 3, 6, and 12 months with total healing and ended splint therapy. A minimum of 6 months was needed for 12 patients to heal and 12 months for an additional 10 patients to heal, with the remaining 3 patients advised to seek advanced surgical treatment. Similarly, Zonnenberg and Mulder [23] instructed their patients to wear the splints for at least 20 hours per day and continued treatment until the remission of TMD symptoms or prior to exceeding 12 months.

The parameter of total healing, as defined above by our study, included diet score, TMD pain, and amount of mouth opening. We researched diet score with splint therapy in our study, differentiating our research from other literature on TMDs. Our results concerning diet scores at the end of 12-month splint therapy included 1 patient (4%) on a soft solid diet and the rest of the 24 patients (96%) able to follow a normal diet. In our literature review, we found that Idle et al. [6] and Leandro et al. [7] examined diet score with TMJ ankylosis and joint replacement. The diets of TMD patients were also surveyed by Haketa et al. [5], who reported that disc displacement TMD groups had worse impairment levels than myofascial disorder patients concerning putting food into their mouths and overall difficulty in consuming a meal, with myofascial disorder patients experiencing relatively less difficulty intaking food. Similar to Haketa et al. [5], our study showed that, at 6 months of splint therapy, patients with disc displacement without reduction had low diet scores (*p*=0.002 < 0.5). Furthermore, Irving et al. [24] reported that, of their 35 patients with temporomandibular disorder pain dysfunction syndrome, 31 of these patients had eating or food intake problems. Raphael et al. [25] also claimed that, in an effort to decrease masticatory activity that exacerbates facial pain, patients with more severe myofascial face pain (MFP) are likely to reduce their intake of dietary fiber. Contrary to Raphael et al. [25], we think that overloading TMJ tissues caused the patients to have low diet scores.

Our study showed muscle pain present in 13 patients in the beginning of splint therapy. At early (3 months) and midterm (6 months) stages of splint therapy, the muscle pain was only eliminated in a total of 7 patients (54%), and an additional 4 patients (9 total; 69%) were pain-free by the end of splint therapy. This means that 31% of myofascial disorder patients with splint therapy have not experienced total pain relief. These results are similar to the findings of Kurita et al. [21] on the effect of splint therapy on muscle pain remission with a 73% success rate, but they did not report on the duration of time splints were used on patients. Kurita et al. [21] and Abekura et al. [15] suggest that patients should wear splints continuously because muscle activities increase if patients stop using the splints. In contrast to this suggestion, our study showed that relative success (69%) of muscle pain relief may not be a result of continuous daily splint use. Similarly, Klasser and Greene [13] also advise against continuous splint use because of irreversible damage to TMJ structures.

There are some limitations of our study like other studies took part in the literature. One limitation of this study is that the work was done retrospectively, analyzing patients' records and files. We evaluated 3 mm splint therapy and its effects on signs and symptoms of TMDs from a distance without control over splint types used (varying thickness or softness of materials) or the use of other conservative treatment options, such as lasers, transcutaneous electrical nerve stimulation (TENS), and self-care counseling. Therefore, our results may provide only unpretending knowledge to the research community due to lacking follow-up data on clinical patients.

## 5. Conclusion

Conclusions drawn from our retrospective research on the effects of 3 mm thick stabilization splints on TMD signs and symptoms are as follows:

1. There were no remarkable effects of splints on total TMD healing in the first 3-month period with a success rate of only 44% (11 patients). There was also no significant difference between 3 months and 6 months (12 patients, 48%).
2. There was no correlation between daily splint usage duration and total healing of TMD signs and symptoms.
3. Researching diet score parameters differentiates our study from other studies on TMDs. In addition, high diet scores were achieved using 3 mm splint therapy.
4. Despite achieving low success rates in early (3 months) and midterm (6 months) treatment periods, by the end of splint therapy (12 months), a success rate of 88% was achieved for 3 mm splint therapy on all symptoms of TMDs, which we consider a successful outcome for a conservative and reversible treatment option.

Finally, TMD signs and symptoms should include TMD pain, amount of mouth opening, and diet score, which encompass our definition of “total healing” of TMDs in our study.

## Figures and Tables

**Table 1:** Statistic calculation of the patient's variables including “diet score” during treatment period.

	VAS	Frequency *n* (%)
*Diet score, month 0*		
Valid	1	6 (24)
	2	16 (64)
	3	3 (12)
	Total	25 (100)
*Diet score, month 3*		
Valid	1	2 (8)
	2	14 (56)
	3	9 (36)
	Total	25 (100)
*p*=0.001		

*Diet score, month 6*		
Valid	1	1 (4)
	2	10 (40)
	3	14 (56)
	Total	25 (100)
*p*=0.012		

*Diet score, month 12*		
Valid	2	1 (4)
	3	12 (48)
	Total System	13 (52) 12 (48)
Missing		
Total	25 (100)	
*p*=0.031		

VAS: verbal scale; *n*: number of patients; *p* < 0.05: statistically significant level.

**Table 2:** Statistic calculation of the patient's variables including “distribution of patients' splint usage duration in a day with respect to total healing” during treatment period.

	*n*	Mean (h)	Std. deviation	Minimum (h)	Maximum (h)
*At month 3*					
No	14	13,21	2,225	11	20
Yes	11	12,27	3,849	8	22
Total	25	12,80	3,014	8	22
*p*=0.045					

*At month 6*					
No	13	14,15	2,794	12	20
Yes	12	12,75	1,815	10	16
Total	25	13,48	2,434	10	20
*p*=0.154					

*At month 12*					
No	3	11,67	4,163	7	15
Yes	10	13,40	1,897	12	18
Total	13	13,00	2,483	7	18
*p*=0.309					

*n*: number of patients; h: hour; *p* < 0.05: statistically significant level.

**Table 3:** Statistic calculation of the patient's variables including TMD pain and muscle pain during the treatment period.

	VAS	Frequency, *n* (%)			Frequency, *n* (%)
*VAS TMD pain score, month 0*			*Muscle pain, month 0*		
Valid	0	3 (12)	Valid	No Yes	12 (48) 13 (52)
	1	7 (28)			
	2	13 (52)			
	3	2 (8)			
	Total	25 (100)		Total	25 (100)
*VAS TMD pain score, month 3*			*Muscle pain, month 3*		
Valid	0	4 (16)	Valid	No	15 (60)
	1	14 (56)		Yes	10 (40)
	2	7 (28)			
	Total	25 (100)		Total	25 (100)
*p*=0.036			*p*=0.043		

*VAS TMD pain score, month 6*			*Muscle pain, month 6*		
Valid	0	9 (36)	Valid	No Yes	19 (76) 6 (24)
	1	9 (36)			
	2	6 (24)			
	3	1 (4)			
	Total	25 (100)		Total	25 (100)
*p*=0.025			*p*=0.037		

*VAS TMD pain score, month 12*			*Muscle pain, month 12*		
Valid	0	9 (36)	Valid	No	9 (36)
	1	4 (16)		Yes	4 (16)
	Total	13 (52)		Total	13 (52)
*p*=0.031			*p*=0.015		

Missing system		12 (48)	Missing system		12 (48)
Total		25 (100)	Total		25 (100)

VAS: verbal scale; *n*: number of patients; *p* < 0.05: statistically significant level.

**Table 4:** Statistic calculation of the patient's variable “distribution of patients' maximal interincisal opening with respect to diagnosis” during the treatment period.

	*n*	Mean	Std. deviation	Minimum	Maximum
*At month 0*					
Without reduction	12	32,33	4,638	27	42
With reduction	13	40,54	2,933	37	45
Total	25	36,60	5,627	27	45
*p*=0.000					

*At month 3*					
Without reduction	12	36,17	3,215	30	43
With reduction	13	40,77	3,140	34	45
Total	25	38,56	3,895	30	45
*p*=0.001					

*At month 6*					
Without reduction	12	38,67	3,367	33	43
With reduction	13	41,46	2,570	37	45
Total	25	40,12	3,244	33	45
*p*=0.028					

*At month 12*					
Without reduction	7	41,00	2,828	37	45
With reduction	6	41,00	1,673	40	44
Total	13	41,00	2,273	37	45
*p*=1.00					

*n*: number of patients; *p* < 0.05: statistically significance level.

**Table 5:** Statistic calculation of the patient's variables including cross-tabulation of total healing data in between treatment periods.

Total healing month 3 ∗ total healing month 6					
			Total healing, month 6		Total
			No	Yes	
Total healing, month 3	No	*n*	12	2	14
	Yes	*n*	1	10	11
Total		*n*	13	12	25
		% within total healing month 3	52.0%	48.0%	100.0%
*p* ≤ 0.001					

Total healing month 3 ∗ total healing month 12					
			Total healing, month 12		Total
			No	Yes	
Total healing, month 3	No	*n*	3	(month 6 + 12) 8 + 3 = 11	14
	Yes	*n*	0	(month 6 + 12) 4 + 7 = 11	11
Total		*n*	3	22	25
		% within total healing month 3	12%	(76.9 + 11.2%) = 88%	100.0%
*p*=0.007					

Total healing month 6 ∗ total healing month 12					
			Total healing, month 12		Total
			No	Yes	
Total healing, month 6	No	*n*	3	4	7
	Yes	*n*	0	6	6
Total		*n*	*n*	10	13
		% within total healing month 6	23.1%	76.9%	100.0%
*p*=0.192					

*n*: number of patients; *p* < 0.05: statistically significant level.

## Data Availability

The data used to support the findings of this study are available from the corresponding author upon request.
